# Effects of High-Intensity Interval Training on Aerobic Capacity in Cardiac Patients: A Systematic Review with Meta-Analysis

**DOI:** 10.1155/2017/5420840

**Published:** 2017-03-12

**Authors:** Bin Xie, Xianfeng Yan, Xiangna Cai, Jilin Li

**Affiliations:** ^1^Department of Cardiology, First Affiliated Hospital of Shantou University Medical College, Shantou, Guangdong 515041, China; ^2^Department of Plastic Surgery, First Affiliated Hospital of Shantou University Medical College, Shantou, Guangdong 515041, China

## Abstract

*Purpose*. The aim of this study was to compare the effects of high-intensity interval training (INTERVAL) and moderate-intensity continuous training (CONTINUOUS) on aerobic capacity in cardiac patients.* Methods*. A meta-analysis identified by searching the PubMed, Cochrane Library, EMBASE, and Web of Science databases from inception through December 2016 compared the effects of INTERVAL and CONTINUOUS among cardiac patients.* Results*. Twenty-one studies involving 736 participants with cardiac diseases were included. Compared with CONTINUOUS, INTERVAL was associated with greater improvement in peak VO_2_ (mean difference 1.76 mL/kg/min, 95% confidence interval 1.06 to 2.46 mL/kg/min, *p* < 0.001) and VO_2_ at AT (mean difference 0.90 mL/kg/min, 95% confidence interval 0.0 to 1.72 mL/kg/min, *p* = 0.03). No significant difference between the INTERVAL and CONTINUOUS groups was observed in terms of peak heart rate, peak minute ventilation, VE/VCO_2_ slope and respiratory exchange ratio, body mass, systolic or diastolic blood pressure, triglyceride or low- or high-density lipoprotein cholesterol level, flow-mediated dilation, or left ventricular ejection fraction.* Conclusions*. This study showed that INTERVAL improves aerobic capacity more effectively than does CONTINUOUS in cardiac patients. Further studies with larger samples are needed to confirm our observations.

## 1. Introduction

Cardiovascular diseases (CVDs) remain the greatest cause of death worldwide. In 2008, more than 17 million people died due to CVDs, of whom 7.3 million died of heart attacks [1]. Interventions are urgently needed to address this worrying trend. CVDs are largely preventable, and cardiac rehabilitation is increasingly recognized as an important component of the continuum of care for patients with coronary artery disease (CAD) and chronic heart failure (CHF). It is included in Class 1 recommendations of the American Heart Association and the American College of Cardiology for the treatment of these patients [2, 3].

According to the World Health Organization, insufficient physical activity is the fourth leading risk factor for mortality, with 6% of deaths worldwide attributed to this factor [1]. Exercise training is essential for cardiac patients. It has an important role in improving endothelial function, which in turn enhances blood flow by causing vasodilatation and improving vasomotor function. Exercise training also contributes to the improvement of many other functions, such as the achievement of good glycemic control and insulin sensitivity, leading to weight loss; the improvement of blood pressure; and the correction of deranged lipid profiles [4, 5]. Proper exercise training is a cost-effective and well-established primary intervention that delays the onset of health burdens associated with various chronic diseases in many cases. The appropriate amount, frequency, and mode of exercise, however, remain unknown. Moreover, the optimum “dose” of exercise to obtain maximum cardiac benefits remains unclear.

Aerobic capacity has been found to be the single best parameter of cardiac function and all-cause death among known cases of CVDs [6]. It is measured directly as peak VO_2_. The improvement of the peak VO_2_ can improve aerobic capacity and promote cardiac rehabilitation. Moreover, reduction of the most common traditional risk factors for CVDs (e.g., hypertension, hyperlipidemia, and obesity) can decrease the occurrence of cardiovascular events. Research suggests that CAD and CHF are associated with impaired endothelial dysfunction, which is evaluated by flow-mediated dilation (FMD) and can be improved through physical exercise [7]. Thus, the identification of more effective exercise programs is needed to improve cardiovascular benefits in cardiac patients.

Moderate-intensity continuous training (CONTINUOUS), a traditional exercise prescription, usually involves walking or cycling for 30–60 min [to reach 40–80% peak oxygen uptake (peak VO_2_)] [8]. However, recent evidence from patients with CHF [9] and CAD [10] suggests that high-intensity interval training (INTERVAL) may be a better modality for the improvement of aerobic capacity. Although INTERVAL has no standard definition, it refers to repeated sessions of brief intermittent exercise, often performed with maximal effort or intensity (i.e., to achieve ≥90% peak VO_2_) [11]. This intensity can be achieved by a single effort lasting a few seconds to several minutes, or with multiple efforts separated by a few minutes of rest or low-intensity exercise. INTERVAL has been shown to have significant benefits, including improved aerobic capacity, endothelial function, and other cardiac functions, in patients with CAD and CHF [12, 13].

Although several reviews and meta-analyses of INTERVAL for CAD and CHF were published [14–16], no consensus has been reached about whether INTERVAL produces superior physical, clinical, and functional benefits compared to CONTINUOUS. We are also unware of any systematic reviews that have assessed the effect of INTERVAL among cardiac patients.

This systematic review was conducted to assess whether INTERVAL produces larger effect sizes for change in aerobic capacity [peak VO_2_, oxygen consumption at anaerobic threshold (VO_2_ at AT), VE/VCO_2_ slope, respiratory exchange ratio (RER), peak minute ventilation (peak V_E_), peak heart rate (PHR)], and physiological and clinical parameters compared with CONTINUOUS among patients with known cardiac disease (including CAD and CHF). The hypothesis of our study was that INTERVAL will have a greater effect on aerobic capacity given the superior improvement in mitochondrial function and cardiac contractility.

## 2. Methods

We conducted this study according to the methods of the Cochrane Handbook for Systematic Reviews of Interventions [17].

### 2.1. Search Strategy

The PubMed, Cochrane Library, EMBASE, and Web of Science electronic databases were searched to identify relevant clinical trials published between the earliest available date and December 2016 using the keywords “heart failure,” “coronary artery disease,” “high intensity interval training,” “interval exercise,” and “high-intensity interval exercise.” The reference lists of retrieved articles were also searched to identify other appropriate studies.

### 2.2. Inclusion and Exclusion Criteria

Only full-text English-language reports of clinical trials were considered for inclusion. In addition, we considered only studies that compared outcomes between an intervention group performing INTERVAL and a control group performing CONTINUOUS, with rhythmic aerobic exercise programs lasting at least 4 weeks. Eligible studies also reported on at least one cardiorespiratory exercise training outcome measure in patients with cardiac disease. Reviews, cases reports, editorial comment, communications, and reports without sufficient data were excluded in our meta-analysis.

### 2.3. Study Selection

**Figure 1:**
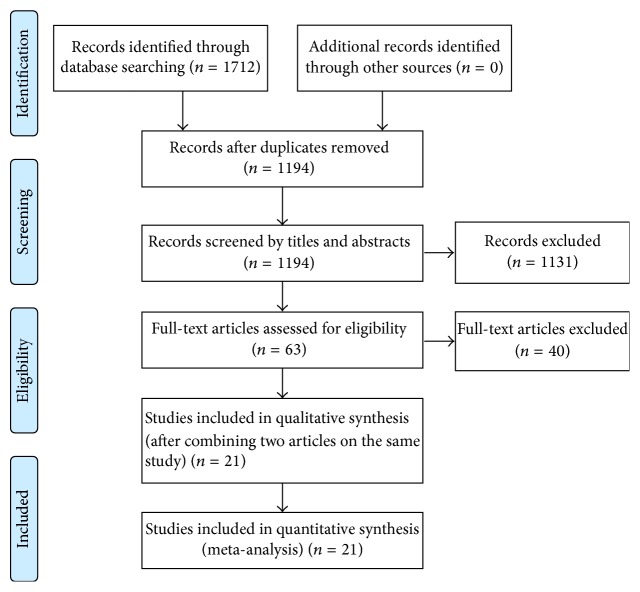
Flow chart of the study selection procedure.

Figure 1 illustrates the flow of study selection. Two reviewers independently screened article titles and abstracts, excluding irrelevant studies. Full texts were then reviewed, and any study not fulfilling the inclusion criteria was excluded. Differences in the assessment of study eligibility were resolved by discussion.

### 2.4. Data Extraction and Management

One reviewer collected the data and the second reviewer rechecked it. Collected data included authors' names, year of publication, country in which the study was conducted, duration of the trial period, participant characteristics, intervention description, and outcomes assessed [peak VO_2_, VE/VCO_2_ slope, RER, peak V_E_, PHR, VO_2_ at AT, body mass, blood pressure, blood lipid parameters, FMD findings, and left ventricular ejection fraction (LVEF)]. Disagreements regarding the data collected were resolved by consensus.

### 2.5. Quality Assessment

The Cochrane collaboration's tool for assessing risk of bias was used for assessing the quality of randomized controlled trials (RCTs) and Physiotherapy Evidence Database (PEDro) scale nonrandomized controlled studies, respectively [17, 18].

### 2.6. Statistical Analysis

The Cochrane Collaboration software (RevMan 5.3; Cochrane Collaboration, Oxford, UK) was used for meta-analyses. We calculated effect sizes by subtracting preintervention from postintervention values. When only baseline and postintervention standard deviations (SDs) were reported, the following formula was used to obtain the missing change value [17]: SD_change_ = √[(SD_pre_)^2^ + (SD_post_)^2^  − 2 × corr(pre, post) ×  SD_pre_ × SD_post_], where corr is the correlation coefficient calculated for each outcome using the formula of Conraads et al. [10]: corr = (SD_pre_^2^ + SD_post_^2^ − SD_change_^2^)/(2 × SD_pre_ × SD_post_). The heterogeneity of included trials was assessed using the *I*^2^ statistic and the chi-squared test for heterogeneity. We used a fixed-effects model for studies showing significant homogeneity (*I*^2^ < 50%) and a random-effects model for other studies. Results were considered significant when *p* < 0.05. To determine the influence of individual studies on the results obtained, we conducted a sensitivity analysis with one-by-one removal of studies. Publication bias was investigated using funnel plots and Egger's regression model.

## 3. Results

### 3.1. Characteristics of Identified Studies

The database search yielded 1712 titles. After the removal of duplicate records and the screening of abstracts and titles to assess relevance, 63 studies were selected for full-text review. After the exclusion of 40 articles which did not comply with the inclusion criteria, the final sample consisted of 23 articles [9, 10, 12, 13, 19–37] that reported on 21 studies. The characteristics of included studies are summarized in Table 1. All included studies were the randomized controlled trials. The 21 studies involved a total of 736 patients (81% male, 19% female) with cardiac disease (eleven studies examined patients with CAD and ten studies examined those with CHF). Four studies were conducted in Norway, three were conducted in Brazil, two each were conducted in the United States, Greece, and Canada, and one each was conducted in the Republic of Korea, Belgium, Netherlands, France, Taiwan, Italy, Spain, and United Kingdom. The duration of training programs ranged from 4 to 24 weeks, and the frequency of exercise training ranged from 2 to 5 days/week.

### 3.2. Risk of Bias

**Figure 2:**
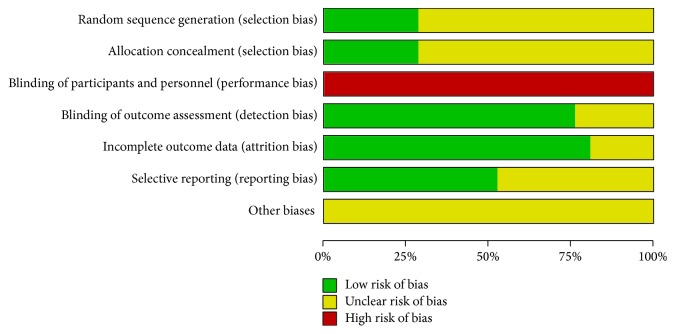
Quality assessment of RCTs using Cochrane collaboration's tool for assessing risk of bias.

Figure 2 shows the risk of bias of the selected studies. Six (28.5%) studies described the methods used to generate and conceal allocation sequences. Participants were not blinded in any study. Outcome assessors were blinded to treatment allocation in sixteen (76.2%) studies. Seventeen (80.9%) studies had incomplete descriptions of outcomes, and eleven (52.3%) studies had low risks of selective reporting bias.

### 3.3. Effects of Interventions on the Cardiorespiratory Measurements

#### 3.3.1. Peak VO_2_

**Figure 3:**
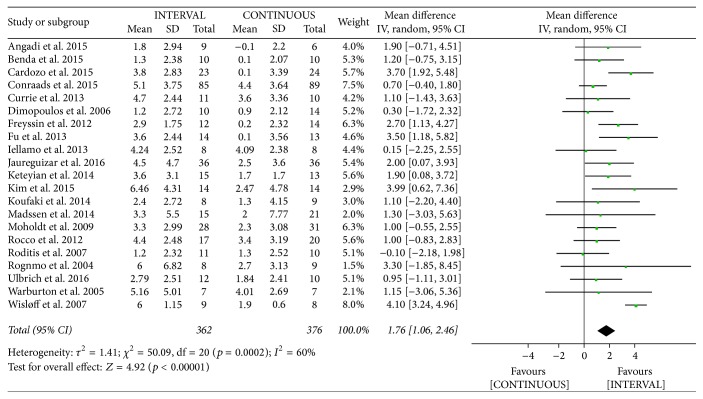
Meta-analysis of effects of INTERVAL on peak VO_2_.

The authors of 21 studies [9, 10, 12, 13, 19–37] involving 738 patients reported on peak oxygen uptake following INTERVAL and CONTINUOUS. Peak VO_2_ improved by 1.76 mL/kg/min [95% confidence interval (CI) 1.06 to 2.46 mL/kg/min] among patients in the INTERVAL groups, which was greater than observed in the CONTINUOUS groups, based on a random-effects model (overall *Z* = 4.92, *p* < 0.001). However, this outcome showed significant heterogeneity (*I*^2^ = 60%, *p* <0.001; Figure 3).

**Figure 4:**
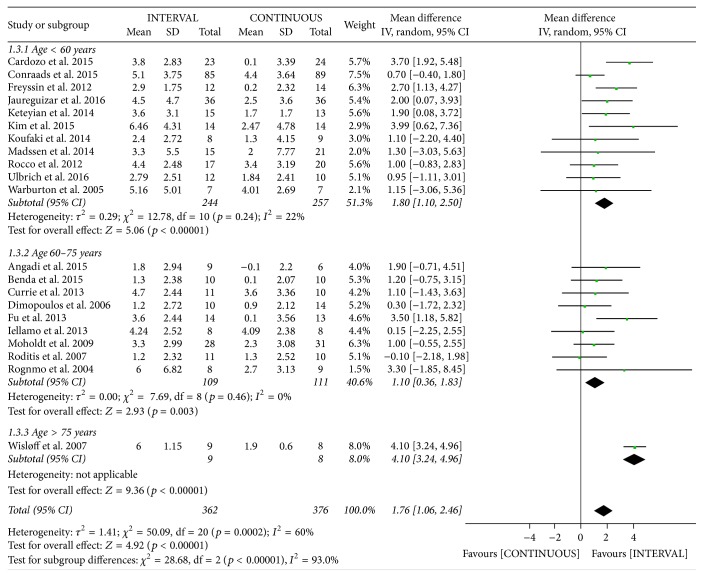
Meta-analysis of the effects of INTERVAL on peak VO_2_ according to age.

**Figure 5:**
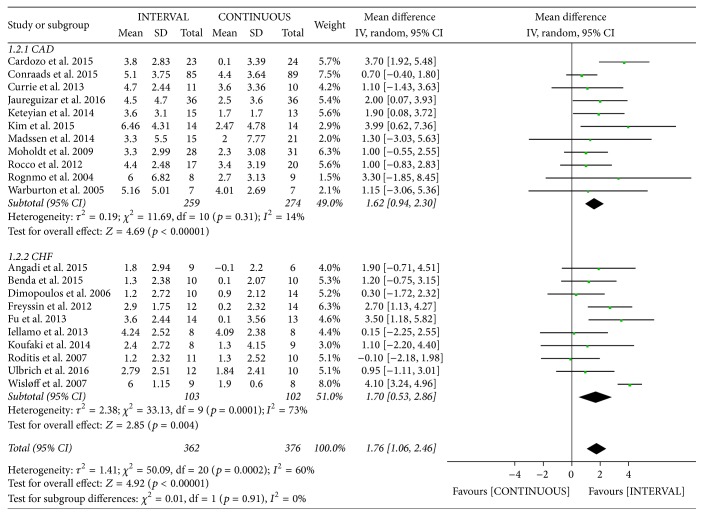
Meta-analysis of the effects of INTERVAL on peak VO_2_ according to disease types.

There was significant heterogeneity in the study outcomes. Therefore, subgroup analysis was performed based on the patient's mean age and disease types. INTERVAL led to significantly greater improvements in peak VO_2_ than did CONTINUOUS in patients aged < 60 years [mean difference (MD) 1.80 mL/kg/min, 95% CI 1.10 to 2.50 mL/kg/min,* p* < 0.001, *I*^2^ = 22%], those aged 61–75 years (MD 1.10 mL/kg/min, 95% CI 0.36 to 1.83 mL/kg/min,* p* = 0.003, *I*^2^ = 0%), and those aged > 75 years (included in only one study [12]; Figure 4). From disease types subgroup analyses, INTERVAL also led to significantly greater improvements in peak VO_2_ than did CONTINUOUS in patients with CAD (MD 1.62 mL/kg/min, 95% CI 0.94 to 2.30 mL/kg/min, *p* < 0.001, *I*^2^ = 14%) and those with CHF (MD 1.70 mL/kg/min, 95% CI 0.53 to 2.86 mL/kg/min, *p* = 0.004, *I*^2^ = 73%; Figure 5).

Sensitivity analysis did not change the statistical significance of the overall results. Exclusion of the study conducted by Wisløff et al. [12], which provided inferior evidence for the effect of INTERVAL on peak VO_2_, significantly improved homogeneity.

#### 3.3.2. VO_2_ at AT

**Figure 6:**
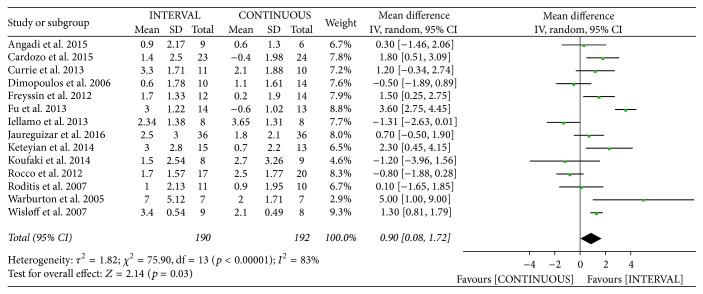
Meta-analysis of effects of INTERVAL on VO_2_ at AT.

The authors of fourteen studies [12, 13, 20, 22–28, 30, 34, 35, 37] involving 382 patients reported on VO_2_ at AT following INTERVAL and CONTINUOUS. VO_2_ at AT improved by 0.90 mL/kg/min [95% CI 0.08 to 1.79 mL/kg/min] among patients in the INTERVAL groups, which was greater than observed in the CONTINUOUS groups, based on a random-effects model (overall* Z* = 2.14,* p* = 0.03). However, this outcome showed significant heterogeneity (*I*^2^ = 83%,* p* < 0.001; Figure 6).

#### 3.3.3. Peak Heart Rate

The authors of seventeen studies [10, 12, 13, 20–23, 25–29, 31, 33, 35–37] involving 611 patients reported on peak heart rate following INTERVAL and CONTINUOUS. A random-effects model revealed no significant difference between groups (MD 0.97 bpm, 95% CI −2.19 to 4.12 bpm, *p* = 0.55).

#### 3.3.4. Peak Minute Ventilation

The authors of five studies [23, 25, 35–37] involving 103 patients reported on peak VE following INTERVAL and CONTINUOUS. A random-effects model revealed no significant difference between groups (MD 3.46 l/min, 95% CI −1.75 to 8.67 l/min, *p* = 0.19).

#### 3.3.5. VE/VCO_2_ Slope

The authors of nine studies [20–23, 26, 28, 34, 35, 37] involving 220 patients reported on VE/VCO_2_ slope following INTERVAL and CONTINUOUS. A fixed-effects model revealed no significant difference between groups (MD 0.24, 95% CI −0.40 to 0.87, *p* = 0.46).

#### 3.3.6. Respiratory Exchange Ratio

The authors of fourteen studies [10, 12, 13, 20, 22, 26–33, 36] involving 579 patients reported on RER following INTERVAL and CONTINUOUS. A random-effects model revealed no significant difference between groups (MD 0.01, 95% CI −0.01 to 0.02, *p* = 0.25).

### 3.4. Effects of Interventions on Physiological and Clinical Parameters

#### 3.4.1. Body Mass

The authors of eight studies [10, 13, 20, 25, 32, 34, 36, 37] involving 363 patients reported decreased body mass following INTERVAL and CONTINUOUS. A fixed-effects model revealed no significant difference between groups (MD 0.55 kg, 95% CI −0.52 to 1.62 kg, *p* = 0.31).

#### 3.4.2. Blood Pressure

The authors of eight studies [9, 10, 13, 20, 25, 27, 28, 36] involving 376 patients reported on systolic blood pressure (SBP) and diastolic blood pressure (DBP) following INTERVAL and CONTINUOUS. A random-effects model revealed no significant difference between groups (SBP: MD −0.09 mmHg, 95% CI −4.82 to 4.65 mmHg, *p* = 0.97; DBP: MD −0.79 mmHg, 95% CI −3.75 to 2.16 mmHg, *p* = 0.60).

#### 3.4.3. Blood Lipids

Data on high-density lipoprotein cholesterol (HDL-C), low-density lipoprotein cholesterol (LDL-C), and triglyceride (TG) levels following INTERVAL and CONTINUOUS were reported in six studies [22, 25, 26, 29, 31, 32] involving 340 patients. A random-effects model showed no significant difference between groups (TG: standardized mean difference (SMD) −0.05, 95% CI −0.26 to 0.17; LDL-C: SMD −0.6, 95% CI −1.3 to 0.11; HDL-C: SMD 0.05, 95% CI −0.17 to 0.26). The result of cholesterol was assessed in four studies [22, 25, 26, 31] involving 253 patients. A random-effects model revealed no significant difference between groups (SMD 0.01, 95% CI −0.55 to 0.57).

#### 3.4.4. Flow-Mediated Dilation

The authors of six studies [10, 12, 13, 20, 21, 31] involving 269 patients reported on FMD following INTERVAL and CONTINUOUS. A random-effects model showed no significant difference between groups (MD 1.47%, 95% CI −0.20% to 3.14%, *p* = 0.09).

#### 3.4.5. Left Ventricular Ejection Fraction

The authors of eight studies [9, 12, 19–21, 25, 26, 32] involving 170 patients reported increased LVEF following INTERVAL and CONTINUOUS. A random-effects model showed no significant difference between groups (MD 2.18%, 95% CI −0.54% to 4.90%, *p* = 0.12).

### 3.5. Publication Bias

Egger's regression analysis excluded relevant publication bias for peak VO_2_ (*p* = 0.14), and the funnel plot of these data was symmetrical.

## 4. Discussion

To our knowledge, most previous systematic reviews on this topic have focused on patients with specific diseases, such as CAD and CHF. One previous review [38] has examined whether INTERVAL is more effective than CONTINUOUS for improving peak VO_2_ and LVEF in CHF patients. However, this review focused only on CHF and only seven articles were included in the review. This systematic review examined the efficacy of INTERVAL as a part of cardiac rehabilitation in patients with cardiac disease (including CHF and CAD). Twenty-one studies involving 738 cardiac patients were included in the review. The main findings were that INTERVAL appears to be at least as effective as and in some cases more effective than CONTINUOUS, for the improvement of aerobic capacity, although we found evidence of heterogeneity among studies. Heterogeneous results for this outcome in the study conducted by Wisløff et al. [12] were due mainly to the inclusion of elderly patients.

### 4.1. Rationale and Potential Working Mechanisms of INTERVAL

Due to repeated alternation of high- and low-intensity exercise, INTERVAL's stimulation of the body fluctuates. The rationale is to accumulate more time in high-intensity zones compared to a continuous exercise where exhaustion would occur more prematurely and therefore to produce a stronger stimulus for cardiovascular and muscular adaptations [39, 40]. The mechanisms involved in the superiority of INTERVAL to CONTINUOUS have not been clearly elucidated. The potential mechanisms for the greater improvement in aerobic capacity achieved by INTERVAL include increased activation of peroxisome-proliferator activated receptor *γ* coactivator (PGC-1*α*), which improves mitochondrial function [12, 41, 42], and increased maximal rate of Ca^2+^ reuptake into the sarcoplasmic reticulum, which reduces skeletal muscle fatigue [12, 42]. The increase in PGC-1*α* to be strongly correlated with the improved VO_2_ peak (*r* = 0.72, *p* < 0.01) was found by Wisløff et al. [12], supporting the influence of mitochondrial function on exercise capacity.

INTERVAL has been demonstrated to activate p38 mitogen-activated protein kinase and 5′-adenosine monophosphate-activated protein kinase. Both of these exercise-responsive signaling kinases are implicated in direct phosphorylation and activation of PGC-1*α*. Increased nuclear abundance of PGC-1*α* following INTERVAL may coactivate transcription factors to increase mitochondrial gene transcription, ultimately resulting in accumulation of more mitochondrial proteins to drive mitochondrial biogenesis [43]. Mitochondrial biogenesis is essential to maintain the structural integrity of skeletal muscle. Mitochondrial function is associated with aerobic physical fitness and plays an important pathophysiological role in cardiac patients. Consequently, the major benefits of INTERVAL interventions include enhanced peripheral blood circulation [44], as well as increased skeletal muscle and functional capacity [45–47]. The improvement of peak VO_2_, a strong, independent predictor of all-cause and cardiovascular-specific mortality [48, 49], through INTERVAL is thus of clinical significance.

The magnitude of difference in the effects of INTERVAL and CONTINUOUS in terms of VE/VCO_2_ slope, RER, peak V_E_, PHR, body mass, blood pressure, blood lipids, FMD, and LVEF was small in the present analysis, which may be related to the examination of short-term outcomes in the included studies. Thus, more research is necessary to provide information on the long-term effects of INTERVAL.

A meta-analysis focused mainly on patients with CHF by Haykowsky et al. [38] showed INTERVAL is more effective than CONTINUOUS for improving peak VO_2_ (MD 2.14 mL/kg/min, 95% CI 0.66 to 3.63 mL/kg/min) but not the LVEF. Another systematic analysis by Smart et al. [50] that analyzed 446 CHF patients revealed that INTERVAL determined a significant increase in peak VO_2_ (MD 1.04 mL/kg/min, 95% CI 0.42 to 1.66 mL/kg/min) and VE/VCO_2_ slope (MD −1.35, 95% CI −2.15 to −0.55). A more recent meta-analysis including CAD patients by Pattyn et al. [51] reported higher increase in VO2 peak with INTERVAL (MD 1.6 mL/kg/min, 95% CI 0.8 to 3.02 mL/kg/min) but VE/VCO_2_ slope, VO2 at AT, and body mass. From our analysis, INTERVAL had similar effect results in improving peak VO_2_ in above meta-analysis. In addition, our systematic review included more evaluative indicators, such as PHR, peak V_E_, VE/VCO_2_ slope, RER, VO_2_ at AT, blood pressure, blood lipids, FMD, and LVEF, than did the previous meta-analysis.

Pooled estimates showed significant heterogeneity among studies included in this review. Important clinical and methodological differences may have affected the results obtained in the intervention and control groups. Some of these differences were in inclusion criteria and among participants, who were in different countries and of different ages.

### 4.2. Study Limitations

Our systematic review has some limitations. Few trials included in the study provided clear descriptions of the randomization and allocation of participants to treatments. Many of the studies failed to describe the blinding of assessors to treatment allocation, which raises the possibility of performance bias. In addition, although we examined publication bias because we searched only four electronic databases, we did not search for unpublished trials. Moreover, the review included only RCTs published in English. Consequently, our results may have been affected by publication bias. Several meta-analyses were affected by statistical heterogeneity, possibly due to differences in study methodologies and data collection techniques (e.g., wide ranges of variability in age, sex, and follow-up duration), which may have affected our findings. Finally, most of the studies had small samples, and no large-scale clinical RCT was included, which likely affected the objectivity and reliability of this meta-analysis and systematic review.

### 4.3. Conclusion

The current analysis indicated that INTERVAL can provide more benefits than CONTINUOUS in terms of improving peak VO_2_ and VO_2_ at AT in patients with cardiac disease. INTERVAL programs, which increase exercise capacity compared with traditional exercise, are thus preferable. Differences in the effects of INTERVAL and CONTINUOUS in terms of PHR, peak V_E_, VE/VCO_2_ slope, RER, body mass, blood pressure, blood lipids, FMD, and LVEF were small and may not be clinically meaningful. The results of this analysis should be interpreted with caution due to the small sample. Accordingly, more high-quality, large-sample, multicenter, long-term randomized interventional studies are needed to assess the effects of INTERVAL in cardiac patients.

## Figures and Tables

**Figure 7:**
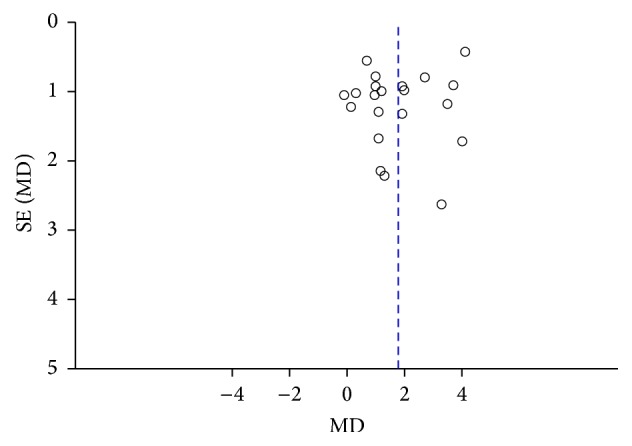
Funnel plot of publication bias.

**Figure 8:**
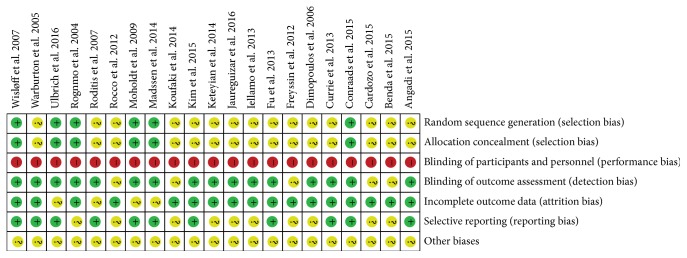
Risk of bias summary.

**Figure 9:**
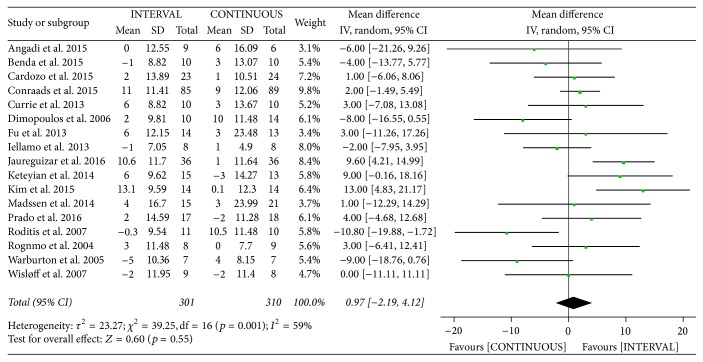
Meta-analysis of effects of INTERVAL on PHR.

**Figure 10:**
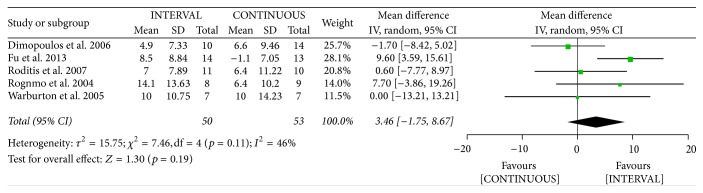
Meta-analysis of effects of INTERVAL on peak VE.

**Figure 11:**
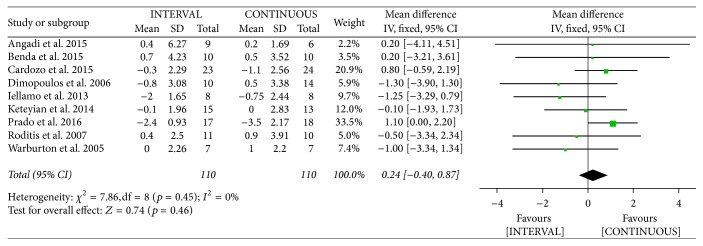
Meta-analysis of effects of INTERVAL on VE/VCO_2_ slope.

**Figure 12:**
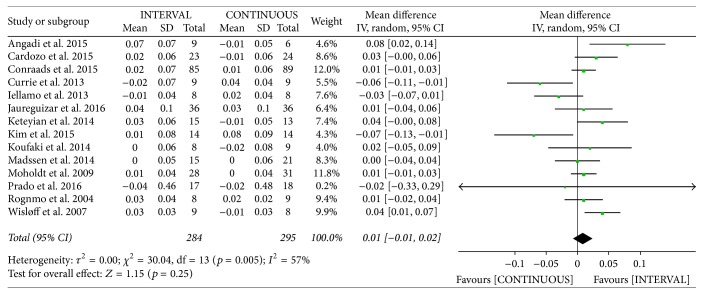
Meta-analysis of effects of INTERVAL on RER.

**Figure 13:**
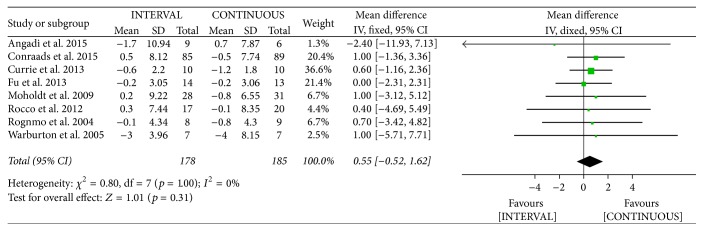
Meta-analysis of effects of INTERVAL on body mass.

**Figure 14:**
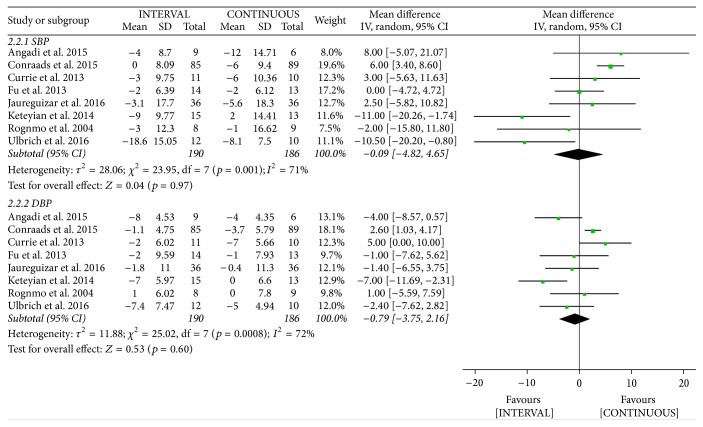
Meta-analysis of effects of INTERVAL on blood pressure.

**Figure 15:**
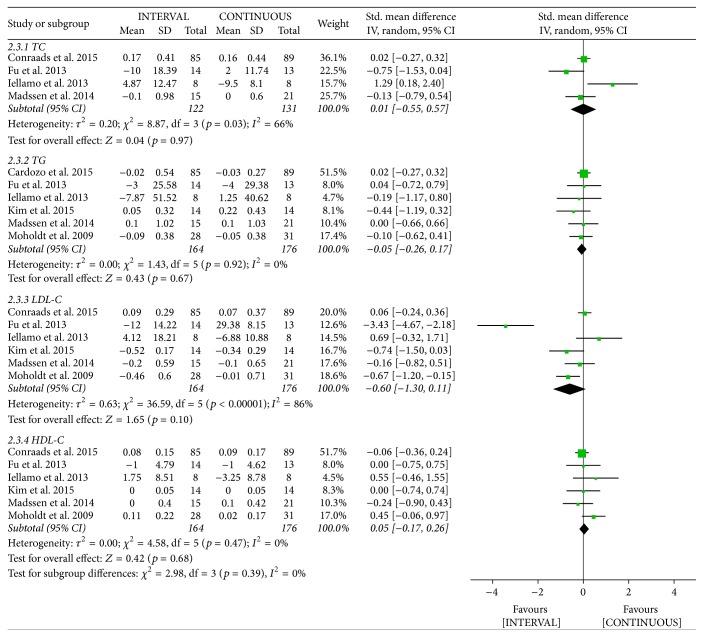
Meta-analysis of effects of INTERVAL on blood lipid.

**Figure 16:**
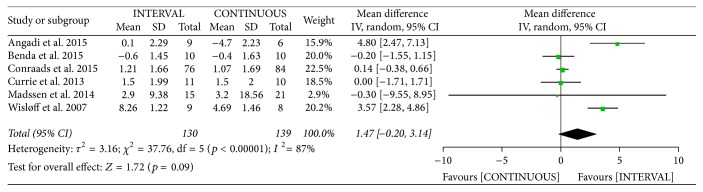
Meta-analysis of effects of INTERVAL on FMD.

**Figure 17:**
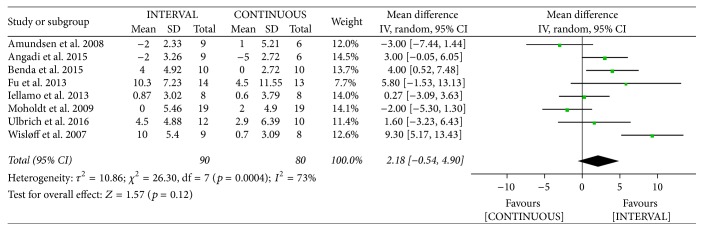
Meta-analysis of effects of INTERVAL on LVEF.

**Table 1:** Characteristics of included studies.

Study	Country	Disease	Patient number		Mean age, year		Mode	Exercise program		Exercise duration (weeks)
			INTERVAL	CONTINUOUS	INTERVAL	CONTINUOUS		INTERVAL	CONTINUOUS	
Angadi et al., 2015	US	CHF	9	6	69	71.5	TM	3 d/wk, 4 × 4 mins @ 85%–90% of peak HR, 3 min recovery	3 d/wk, 30 mins @ 70% of peak HR	4

Benda et al., 2015	Netherlands	CHF	10	10	63	64	Cycling	2 d/wk, 3.5 × 1 min @ 90%, of maximal workload, 10 periods	2 d/wk, 30 min @ 60%–75% of maximal workload	12

Cardozo et al., 2015	Brazil	CAD	23	24	56	62	TM	3 d/wk, 2 mins @ 90% of peak HR 2 min recovery @ 60% peak HR	3 d/wk, 30 mins @ 70%–75% of peak HR	16

Conraads et al., 2015	Belgium	CAD	85	89	57	59.9	Cycling	3 d/wk, 4 × 4 mins @ 90–95% of peak HR, 3 min recovery. 38 min total	3 d/wk, 37 mins @ 70–75% of peak HR. 47 min total	12

Currie et al., 2013	Canada	CAD	11	11	62	68	Cycling	2 d/wk, 10 × 1 min @ 80%–104% PPO, 1 min recovery@10% PPO	2 d/wk, 30–50 min @ 51%–65% PPO	12

Dimopoulos et al., 2006	Greece	CHF	10	14	59.2	61.5	Cycling	3 d/wk, 30 s@100% of WRp, 30 s rest, 40 min total	3 d/wk, 40 mins @ 50% of WRp	12 (36 sessions)

Freyssin et al., 2012	France	CHF	12	14	54	55	Cycling	5 d/wk, 12 × 30 s @ 50% (4 wks) + 80% (4 wks) of maximum power, 1 min recovery @ rest	5 d/wk, 45 mins @ HRVT1	8

Fu et al., 2013	Taiwan	CHF	14	13	67.5	66.3	Cycling	3 d/wk, 5 × 3 mins @ 80% of peak VO_2_ 3 min recovery @ 40% peak VO_2_	3 d/wk, 60 mins @ 60% of peak VO_2_	12

Iellamo et al., 2013	Italy	CHF	8	8	62.2	62.6	TM	2–5 d/wk, 2–4 × 4 mins @ 75%–80% of HHR 3 min recovery @ 45%–50% of HHR	2–5 d/wk, 30–45 mins @ 45%–60% of HHR	12

Jaureguizar et al., 2016	Spain	CAD	36	36	58	58	TM	(15–30) × 40 s @ the first (second) steep ramp test	15–30 mins @ VT1	8

Keteyian et al., 2014	US	CAD	15	13	60	58	TM	3 d/wk, 4 × 4 mins @ 80%–90% of HRR, 3 min recovery @ 60%–70% of HRR	3 d/wk, 30 mins @ 60%–80% of HRR	10

Kim et al., 2015	Republic of Korea	CAD	14	14	57	60.2	TM walking	3 d/wk, 4 × 4 mins @ 85%–95% of HRR, 3 min recovery @ 450%–70% of HRR. 5 min total	3 d/wk, 25 mins @ 70%–85% of HRR. 45 min total	6

Koufaki et al., 2014	UK	CHF	9	8	59.8	59.7	Cycling	3 d/wk, 2 × 10 min @ 100% of PPO, 5 min recovery @ 20%–30% of PPO	3 d/wk, 1–4 months: 3 × (7–10) mins; 5-6 months: 40 mins; @ 40%–60% of peak VO_2_	24

Madssen et al., 2014	Norway	CAD	15	21	55.5	60.5	TM	3 d/wk, 34 × 4 mins @ 85%–95% of peak HR, 3 min recovery @ 70% of peak HR	3 d/wk, 46 mins @ 70% of peak HR	12

Moholdt et al., 2009	Norway	CAD	28	31	60.2	62	TM	5 d/wk, 4 × 4 mins @ 90% of peak HR, 3 min recovery @ 70% peak HR, 40 min total	5 d/wk, 46 mins @ 70% of peak HR	4

Rocco et al., 2012^*∗*^ Prado et al., 2016^*∗*^	Brazil	CAD	17	20	56.5	62.5	TM	3 d/wk, 7 × 3 mins @ RCP, 7 × 3 min @ VAT. 42 min total	3 d/wk, 50 mins @ VAT	12

Roditis et al., 2007	Greece	CHF	11	10	63	61	Cycling	3 d/wk, 30 × 30 s @ 100% of WRp, 40 min total	3 d/wk, 40 mins @ 50% of WRp	12 (36 sessions)

Rognmo et al., 2004^*∗*^ Amundsen et al., 2008^*∗*^	Norway	CAD	8	9	62.9	61.2	TM walking	3 d/wk, 4 × 4 mins @ 85%–95% of peak HR, 3 min recovery. 33 min total	3 d/wk, 41 mins @ 50%–60% of peak VO_2_	10

Ulbrich et al., 2016	Brazil	CHF	12	10	53.1	54	TM	3 d/wk, (4–6) × 3 mins @ 95% of peak HR, 3 min recovery @ 70% peak HR. 60 min total	3 d/wk, 30 mins @ 75% of peak HR. 60 min total	12

Warburton et al., 2005	Canada	CAD	7	7	55	57	TM	2 d/wk, 2 mins @ 90% of VO_2_ reserve, 2 min recovery. 30 min total	2 d/wk, 30 mins @ 65% of VO_2_ reserve	16

Wisløff et al., 2007	Norway	CHF	9	9	76.5	74.4	TM	3 d/wk, 4 mins @ 90%–95% of peak HR, 3 min recovery @ 50%–70% peak HR. 38 min total	3 d/wk, 47 mins @ 70%–75% of peak HR	12

INTERVAL, high-intensity interval training; CONTINUOUS, moderate-intensity continuous training; CHF, chronic heart failure; CAD, coronary artery disease; TM, treadmill; HR, heart rate; PPO, peak power output; HRR, heart rate reserve. WRp, 100% peak work rate. VAT, ventilatory anaerobic threshold. VO_2_, oxygen uptake. ^*∗*^means two articles on the same study.

## Appendix

See Figures 7–17.
